# Cohort Profile: Nausea and vomiting during pregnancy genetics consortium (NVP Genetics Consortium)

**DOI:** 10.1093/ije/dyv360

**Published:** 2016-02-26

**Authors:** Lucía Colodro-Conde, Simone M Cross, Penelope A Lind, Jodie N Painter, Annika Gunst, Patrick Jern, Ada Johansson, Merete Lund Maegbaek, Trine Munk-Olsen, Dale R Nyholt, Juan R Ordoñana, Lavinia Paternoster, Juan F Sánchez-Romera, Margaret J Wright, Sarah E Medland

**Affiliations:** 1 QIMR Berghofer Medical Research Institute, Brisbane, Australia,; 2 Department of Human Anatomy and Psychobiology, University of Murcia, and IMIB-Arrixaca, Murcia, Spain,; 3 Department of Psychology, Åbo Akademi, University, Turku, Finland,; 4 Department of Psychology and Speech-Language Pathology University of Turku, Turku, Finland,; 5 National Center for Register-based Research, Aarhus University, Aarhus Denmark,; 6 Institute of Health and Biomedical Innovation, Queensland University of Technology, Brisbane, Australia,; 7 MRC Integrative Epidemiology Unit, School of Social & Community Medicine, University of Bristol, Bristol, UK,; 8 Department of Developmental and Educational Psychology, University of Murcia and IMIB-Arrixaca, Murcia, Spain and; 9 Queensland Brain Institute, University of Queensland, Brisbane, Australia

## Why was the cohort consortium set up?

Nausea and vomiting during pregnancy (NVP), commonly known as morning sickness, is very common and is typically self-limiting. However, more severe forms and the development of hyperemesis gravidarum (HG), defined as persistent and excessive vomiting, with dehydration, ketonuria and >5% bodyweight loss,^1^ may lead to health consequences for the mother and the offspring exposed *in utero.* Despite efforts towards understanding the causes of NVP and HG, they are not well established. The NVP Genetics Consortium is an open collaborative network of researchers integrating data on NVP of women who have been pregnant at least once, with the goal of investigating NVP, NVP severity and HG. Currently, the NVP Genetics Consortium brings together data from Australia, Finland, Spain, the UK and Denmark. The Consortium is actively recruiting new members.

Early consortium efforts started at the QIMR Berghofer Medical Research Institute (QIMR), Brisbane, Australia, including the collection of data in three different samples from ongoing studies: the QIMR NVP study, the Australian Endogene Study (QIMR AES) and the QIMR Mothers of Twins (QIMR MT) study. The QIMR NVP study was specifically designed as a comprehensive data collection on NVP. The two other studies asked questions about NVP as part of other health-related projects. In 2013, the Genetics of Sexuality and Aggression twin samples (GSA, Åbo Akademi University in Finland) and the Murcia Twin Registry (MTR, University of Murcia in Spain), introduced questions on NVP in their protocols with the aim of participating in this project. In 2014, the Avon Longitudinal Study of Parents and Children (ALSPAC, University of Bristol in England), and in 2015, the Danish National Hospital Register (DNHR, in Denmark) began collaborating with data on NVP and HG that were already available. Currently, each study group has independent funding and shares summary statistics on the questions of interest.

Meta-analysis has shown that around 70% of pregnant women worldwide experience some symptoms of NVP^2^ with different degrees of severity. It is estimated that 1.1% of women suffer HG. A recent systematic review highlights that mild/moderate NVP is associated with favourable fetal outcome in terms of decreased risk of miscarriages, congenital malformations, prematurity and developmental achievements.^3^ However, NVP affects the physical health of pregnant women and can also affect their psychosocial functioning, with consequences in both personal and professional domains^4^ that may result in a lower health-related quality of life.^5^ Severe NVP and HG are risk factors for postnatal depression.^6^^,^^7^ NVP and HG also entail economic costs, estimated at almost 1.8 billion USD in the USA in 2012.^8^

The aetiology of NVP appears to be multifactorial. Several lines of evidence support a genetic predisposition to NVP and HG.^9–11^ There is one published twin study on the topic, conducted in the population-based Norwegian Twin Panel,^9^ showing higher correlations of occurrence of NVP and taking nausea medication during pregnancy for monozygotic twins than for dizygotic twins. Genetic variants influencing the human chorionic gonadotrophin hormone, serotonin and autoimmune functioning have been proposed as candidates for NVP.^12^^,^^13^ It has also been proposed that there is a higher frequency of severe NVP in patients with disorders in taste sensation, in the glycoprotein hormone receptor or in fatty acid transport.^14–18^

The general aim of the NVP Genetics Consortium is to add knowledge to the characterization of the genetic, but also the environmental, risk factors for HG as well as NVP occurrence and severity, in order to facilitate development of novel therapeutics and more individualized health interventions. Our specific objectives are: (i) to increase the knowledge of the relative impact of genetic vs environmental causes of HG, NVP and NVP severity; (ii) using genome-wide association studies (GWASs), to identify genetic or genetically influenced risk factors for these phenotypes; and (iii) to elucidate the socio-demographic, health- and lifestyle-related factors accounting for individual differences in these disorders, with special attention to psychological variables.

To address the first of our aims, the two twin samples that are part of the Consortium will conduct a joint twin analysis to estimate the heritability and the genetic correlation between phenotypes related to NVP. Large sample sizes, assembled by the Consortium, will enable us to search for genetic variants involved in NVP/HG using GWAS^19^ and bioinformatic analyses of pathway and regulatory networks will clarify the relationships between candidate and novel genetic risk factors. Meta-analysis will allow us to maximize power while explicitly checking for the presence of latent population substructure/ethnicity or sample/genotype based artefacts through heterogeneity tests. Likewise the combined summary statistics will allow us to maximize power for multivariate linkage disequilibrium (LD) score regressions to estimate the extent to which the genetic correlation between NVP traits can be explained by common variants.^20^ The third of our questions will be addressed with cross-sectional analyses among cohorts that will examine social risk factors and epidemiological data. Additionally, the twin studies will estimate the amount of variance explained by psychosocial and environmental risk factors. To aid in the interpretation of significant results, targeted follow-up analyses will examine the effect of parity and potential covariates.

We expect our collaborative work will result in a better prediction of which women will be at risk for NVP and HG and the development of more effective interventions along with treatments that will directly prevent the progression from moderate to severe NVP or HG.

## Who is in the cohort?

Currently, the NVP Genetics Consortium incorporates data on approximately 13 640 women reporting NVP and 3116 reporting no experience of NVP from the Australian, Finnish, Spanish and British samples, and around 9074 women suffering from HG as recorded by the Danish Register within a population-based cohort of 565 486 women (the reminder of which will serve as controls). Differences in data collection across the cohorts have led to the adoption of broad phenotypic definitions (e.g. experiencing NVP or not). With the exception of QIMR NVP, all the samples are unselected with regards to the phenotype of interest. Two of the studies (Finnish and Spanish) are twin cohorts. Sample sizes, age at the time of the survey and a summary of the availability of the main variables on NVP/HG collected across the cohorts, are provided in Table 1.

**Table 1.** Basic demographics and availability of main variables on NVP and HG data across current participating cohorts in the NVP Genetics Consortium

Study	*n*	Age at survey time [M (SD), range]	Number of children [M (SD), range]	NVP Occurrence	NVP severity	NVP duration	NVP medication	NVP visits to hospital	HG	Sex	Pregnancy order	Age at pregnancy	Singleton/ twin pregnancy	Genotyped (sub) samples	DNA samples available
QIMR NVP	933	32.53 (7.21), 19–75	1.72 (1.07), 0–7	X	X	X	X	X	X	X	X	X	X		
QIMR AES	1400	48.23 (8.68), 26–78	1.96 (1.05), 0–6	X	X	X	X	X			X			X	
QIMR MT	296	45.85 (4.75), 30–59	3.20 (1.04), 2–8	X	X	X	X	X			X		X	X	
GSA	1368	34.63 (7.12), 25–56	1.75 (1.22), 0–12	X	X	X	X	X			X	X	X	X^c^	X
MTR	551	56.54 (7.39), 47–73	2.54 (1.10), 1–8	X	X	X	X	X			X			X^d^	X
ALSPAC^a^	12 208	28.21 (4.87), 15–44		X		X	X			X	X	X	X	X	
DNHR^b^	565 486	29.39 (4.93), 13–55						X	X	X	X	X	X	X	

QIMR AES, QIMR Australian Endogene Study; QIMR MT, QIMR Mothers of Twins; GSA, Genetics of Sexuality and Aggression; MTR, Murcia Twin Registry; ALSPAC, Avon Longitudinal Study of Parents and Children; DNHR, Danish National Hospital Register.

^1^Age at time of birth of the child (1991–92).

^2^Age corresponds to the first pregnancy with records on excessive vomiting.

^3^At present, approximately 130 single nucleotide polymorphisms (SNPs) have been genotyped.

^4^At present, 66 SNPs have been genotyped.

The QIMR NVP study was specifically launched for a comprehensive data collection on NVP through an online survey, starting in 2013 and currently ongoing. It follows a self-selected sampling strategy to collect data from women who have suffered from NVP as inclusion criterion. This strategy over-samples cases in order to create an extreme study design. Currently, 933 women have completed the survey and the goal is to reach data of around 1300 women with severe NVP. We aim to collect 1000 saliva samples in a second stage of this study. Women are being recruited via media appeals and postings to pregnancy/parenting forums. The second QIMR Berghofer sample, QIMR AES, consists of 1400 women who were approached for a larger study on common health conditions. In 2012, women previously recruited for a study on endometriosis^21^ were contacted by letter or email and invited to complete an online questionnaire, including questions about NVP. The response rate was 81%. The QIMR MT cohort comprises the mothers of adolescent twins who are taking part in the Brisbane Adolescent Twin Study. The mothers are approached when the twins are assessed at QIMR Berghofer. The response rate is ∼97% and, to date, 296 women have participated. Of these participants, 90.54% have also given birth to singletons, so the QIMR MT sample confers an opportunity to study in more depth the development of NVP in twin pregnancies, which are risk factors for both NVP and HG,^22–24^ and the experience of NVP in singleton pregnancies in the same women.

The GSA project consists of a large, population-based twin cohort launched at the Abo Akademi University (Finland), first assembled in 2005–06 (see refs^25^^,^^26^ for a detailed description of the original data collections). Data on NVP were collected in 2013 from a subsample of female twins and female siblings of twins, which represents a longitudinal data collection—although longitudinal data arecurrently not available for NVP. The subsample included in the NVP Genetics Consortium consists of 1368 women who have reported being pregnant at least once, with 296 being an MZ twin (41 complete pairs), 587 a DZ twin (313 are DZ same-sex twins, 37 DZ complete pairs), 25 twins of undefined zygosity and 460 sisters of twins. Data were collected in 2013 by a secure, online questionnaire, and zygosity was determined by genotyping and questionnaire. Response rate to the 2013 questionnaire was 43.5%.

The MTR sample is population-based and consists of all twin pairs born between 1940 and 1966, with administrative residence in the Region of Murcia, in Southeast Spain (MTR; see refs^27^^,^^28^). For the NVP Genetics Consortium, the MTR has provided data on 551 women consisting of 294 from MZ twin pairs and 257 from DZ same-sex twin pairs. The response rate in this sample was close to 75%. Zygosity was determined by questionnaire and DNA testing, using short tandem repeat approaches based on 14 autosomal loci plus amelogenin gender determination. Data on NVP were collected in 2013 by telephone interview.

The ALSPAC sample comprises 12 208 women with data available on NVP, representing 83.96% of the original study (for more details of the cohort, see ref.^29^). Eligible women were all those who were pregnant and resident in a defined area in South West England, with an expected date of delivery between 1991 and 1992. The study website contains details of all the data that are available through a fully searchable data dictionary [http://www.bris.ac.uk/alspac/researchers/data-access/data-dictionary]. Data were collected through self-report questionnaires, with NVP data collected on up to four occasions during the index pregnancy (at enrolment and at 18 and 32 weeks of gestation) and 8 weeks after birth.

Lastly, the DNHR^30^ was initiated in 1977 and holds information on all treatments at somatic facilities in Denmark. Information on inpatient treatment is available since 1977 and on outpatient treatment since 1995. The register holds information on all women treated specifically for HG in pregnancy (ICD-10 code: O21), as well as information on gestational age and other pregnancy-related diagnoses. Additional relevant information was drawn from the Danish Civil Registration System.^30^ Women included in the present study were born after 1955, and gave birth to a live-born child between January 1995 and June 2012. Women who had records of HG contributed with information for the first pregnancy/childbirth with HG diagnoses, which was not necessarily the first pregnancy ever.

## How often have they been followed up?

The aim of the NVP Genetics Consortium is to bring together the effort and resources of different research groups who are willing to investigate the proposed research questions. For this reason, follow-ups will be possible in only some of the cohorts that are participating, which at the moment are subsets of women of the QIMR NVP and QIMR MT cohorts. Specifically, women of the QIMR NVP reporting severe NVP or HG will be invited to answer more in-depth questions and to provide saliva samples for genotyping in the present year. A subset of the women of the QIMR MT are being contacted again 2 years later, which will allow checking of the consistency of retrospective reporting; currently their answers regarding NVP do not show any discordance.

## What has been measured?

The most extensive information on NVP is provided by the QIMR NVP study, which includes details on severity, duration and impact for each pregnancy, putative risk factors, medication use and effectiveness. For each pregnancy, participants are asked to rate their degree of NVP using qualifiers including the number of days with NVP during the pregnancy, impact on normal daily routine, consultation with medical professionals, prescription of medication, use of nutritional support and weight loss. In the other two QIMR samples, data on occurrence of NVP, duration and severity according to the same seven-point scale were collected for the first pregnancy, last pregnancy and others. For the GSA and MTR samples, women responded to questionnaires regarding different aspects of their health and sexual and reproductive history, including whether they had suffered from nausea and vomiting during any of their pregnancies. Subsequent details about NVP were collected with reference to the most affected pregnancy, and included information about the trimester(s) in which the symptoms took place as well as their severity, following the method of Zhang *et al.*^11^

Women in ALSPAC provided information while pregnant about their current pregnancy in the context of questionnaires and clinic visits. During their pregnancies, these women were asked whether they were suffering from NVP and if they were using medication for it. Women in the DNHR have records of all in- and outpatient visits in the Danish Hospital system, which can be linked to the ICD-10 codes and socioeconomic variables.

Women of QIMR AES, QIMR MT, ALSPAC and DNHR have existing genome-wide data and most women from QIMR NVP will have it after the first follow-up. GSA and MTR have collected DNA and some single nucleotide polymorphisms have been genotyped.

Data in all samples were based on self-report and were retrospective for the Australian, Spanish and Finnish samples; data from ALSPAC and DNHR women were collected prospectively. NVP presence, severity and duration from the most affected pregnancy will be the primary phenotypes used in the consortium analyses. We will use meta-analysis to combine data across studies with heterogeneous phenotypes, to improve power to detect novel associations. Other lifestyle and health variables have been collected by these cohorts and will be used for specific/secondary analyses.^25^^,^^27–31^

## What has the NVP Genetics Consortium found?

The NVP Genetics Consortium has showed heritability estimates of 73% (95% CI 57–84%) for occurrence, 51% (95% CI 36–63%) for duration and 53% (95% CI 38–65%) for severity of NVP.^32^ These results are consistent with those of the Norwegian Twin Panel^9^ although not reported in their paper; using the tetrachoric correlations and sample sizes, we used the Mx program^33^ to estimate heritabilities of 53% for NVP and 73% for taking nausea medication.

Epidemiological analysis of the data offers some information that is consistent with previous studies. Table 2 summarizes data on the NVP severity in a five-point scale, the trimester when NVP was reported and the visit to the hospital because of this problem, when available. It includes only those women with at least one complete pregnancy.

**Table 2.** NVP severity, trimester in which NVP occurred and attendance at hospital because of NVP (valid %, *n*), segregated by sample

NVP	QIMR NVP^a^ (*n* = 879)	QIMR AES^a^ (*n* = 1273)	QIMR MT (twin data) (*n* = 296)	QIMR MT (singleton pregnancy)^a^ (*n* = 268)	GSA^b^ (*n* = 1181)	MTR^b^ (*n* = 551)	ALSPAC^c^ (*n* = 12 208)	DNHR^d^ (*n* = 565 486)
NVP severity								

No NVP/NVP < 7 days, minor impact	4.8 (39)	47.1 (599)	53.4 (158)	67.2 (180)	40.1 (473)	46.1 (250)	13.08 (1597)^e^	
NVP 7 + days, no medical consultation, minimal impact	9.2 (74)	10 (127)	19.3 (57)	15.7 (42)	30.7 (362)	25.8 (140)		
NVP 7 + days, no medication, minor role impairment	19.4 (156)	23.1 (294)	20.6 (61)	13.4 (36)	25.2 (298)	7 (38)		
Medication, no weight loss, moderate role impairment	15.7 (126)	10.3 (131)	2.7 (8)	1.7 (5)	2.3 (27)	16.2 (88)		
Medication/IV/feeding tube, weight loss, major role impairment	50.9 (410)	9.6 (122)	4.1 (12)	1.7 (5)	1.8 (21)	4.8 (26)		

NVP during pregnancy								

1st trimester	82 (721)	98.4 (665)	97.1 (134)	96.6 (85)	95.1 (673)	97 (290)	74.5 (8969)	
2nd trimester	69.6 (612)	68.2 (461)	52.2 (72)	33 (29)	48.7 (345)	43.1 (129)	47.5 (5040)	
3rd trimester	48.4 (425)	38.1 (257)	20.3 (28)	8 (7)	12.1 (86)	29.4 (88)	27.8 (2946)	
NVP use of health services	22.6 (199)	21.1 (142)	4.7 (14)	1 (3)	7.5 (53)	7 (20)		1.6 (9074)

QIMR AES, QIMR Australian Endogene Study; QIMR MT, QIMR Mothers of Twins; GSA, Genetics of Sexuality and Aggression; MTR, Murcia Twin Registry: ALSPAC, Avon Longitudinal Study of Parents and Children; DNHR, Danish National Hospital Register.

^a^Maximum scores reported across pregnancies.

^b^Reported most affected pregnancy.

^c^Present pregnancy.

^d^First pregnancy with excessive NVP.

^e^ALSPAC women reported if they had suffered NVP, regardless of the severity. For this reason, this proportion could be underestimated (it does not include women with NVP for less than 7 days). A total of 10 611 women (87%) reported NVP.

The generalizability of the results should be interpreted in the context of each sample. In line with previous studies,^34^ around 50% of the participants in the unselected samples reported experiencing NVP of more than 7 days which resulted in a disruption of their daily routine during at least one of their pregnancies. However, the percentage was higher (95.2%) for women of the QIMR NVP sample, which was designed to over-sample NVP cases. The prevalence was also higher in ALSPAC (86.2%), where participants were asked if they had experienced any nausea and vomiting, with no duration or severity specified. In addition, between 74.5% and 98.4% of the participants with NVP reported experiencing NVP in the first trimester, between 33% and 69.6% reported NVP in the second trimester and between 8% and 48.4% in the third trimester. With regard to the use of health services, 1–22.6% of participants presented to hospital because of NVP.

**Figure 1:**
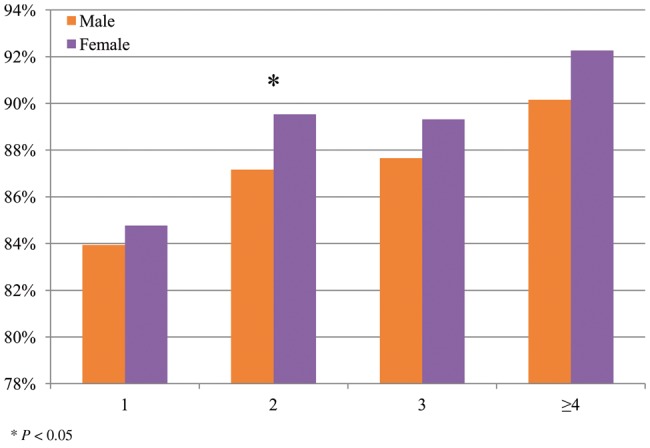
Occurrence of NVP across pregnancies, according to sex of the baby (n = 11 797 singleton pregnancies from ALSPAC sample).

Using data from the ALSPAC sample, excluding twin pregnancies, we examined the relationship between the occurrence of NVP, sex of the baby and pregnancy order. As shown in Figure 1 (Occurrence of NVP across pregnancies, according to sex of the baby, n = 11 797 singleton pregnancies from ALSPAC sample), NVP is more prevalent as the number of pregnancies increases and in female pregnancies (87.7%) vs male pregnancies (86.1%), odds ratio (OR) = 1.14 (95% CI: 1.02–1.27, *P* = 0.02). These results were consistent with those of other cohorts. For women from the QIMR NVP cohort, 90.08% female pregnancies and 89.9% male pregnancies were affected by NVP. However, since women from this cohort were selected to over-sample cases, the results may not be generalizable. Additionally, overall 1.6% women from the DNHR had at least one record of excessive vomiting during pregnancy (O21 codes in the ICD-10). Among these the prevalence was 1.8% among pregnancies with a female fetus vs 1.4% with a male fetus, OR = 1.30 (95% CI: 1.25–1.36, *P* < 0.0001).

Data from the QIMR MT sample showed that women were more likely to suffer from NVP for more than 7 days during twin pregnancies (46.62%) as compared with other reported pregnancies (32.79%), OR = 1.79 (95% CI: 1.27–2.52, *p* = 0.0009). Among those women from the QIMR NVP cohort with twin pregnancies, however, there was no difference in the prevalence of NVP in twin pregnancies (92.45%) vs singleton pregnancies (81.63%), OR = 2.76, 95% CI: 0.92–8.3, *P* = 0.07. According to records of the DNHR, the prevalence of pregnancies presenting with an ICD-10 diagnosis related to excessive vomiting was twice as frequent in twin pregnancies (3.0%) as compared with singleton pregnancies (1.6%), OR = 1.92 (95% CI: 1.72–2.13, *p* < 0.0001). Data from ALSPAC show no difference in the percentage of symptoms of NVP in women having twins (91.7%) compared with those having singletons (86.6%), OR = 1.71, 95% CI: 0.97–3.03, *p* = 0.06.

**Figure 2:**
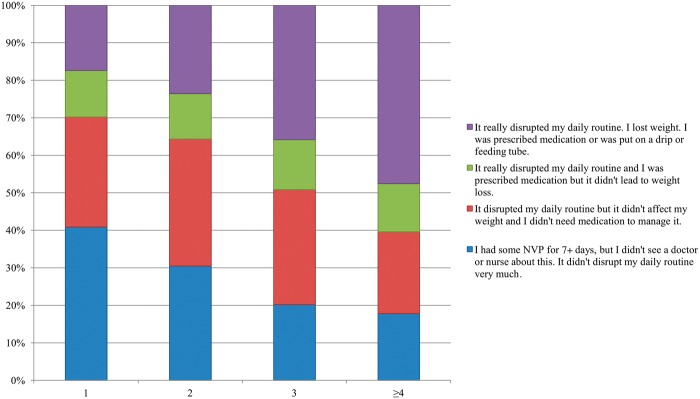
NVP severity for the most affected pregnancy reported by birth order, including data from QIMR NVP, GSA and MTR (n = 1478).

Figure 2 (NVP severity for the most affected pregnancy reported by birth order, including data from QIMR NVP, GSA and MTR, n = 1478) shows the severity of NVP of the most affected reported pregnancy for women in the GSA, MTR and QIMR NVP samples. Not only the occurrence, but also the severity, of NVP is higher as the number of pregnancies increases. All of these results are similar to those of other studies.^22^^,^^24^^,^^35^

## What are the main strengths and weaknesses?

The collaboration between the researchers in the NVP Genetics Consortium confers an exceptional possibility to conduct a systematic examination of the aetiology of NVP and HG in large samples. Many of these cohorts have existing genome-wide data or DNA, which will allow us to conduct the first GWAS for these traits. Since the participants come from different cohorts, it will be possible to analyse environmental risk factors specific to each sample. Moreover, the most significant work to date on the genetics of NVP and HG^9–11^ has focused on case/control style analyses. The data currently available within the Consortium will also allow examination of the high variability, the complexity and the psychosocial correlates and consequences of these conditions.

There are some limitations that will need to be taken into account. For instance, there may be recall bias in those women reporting data about NVP from pregnancies that took place some years ago. As many of the participants within the cohorts are within child-bearing age, it is possible that we may be underestimating prevalence and severity as there is the potential for more affected pregnancies, and few studies have followed up participants who experienced early pregnancy loss. Because the Consortium is using existing data from existing studies, not all variables are available for all studies. The generalizability of some of the results derived from the Consortium may be limited to the unselected samples. Last, historical and social background should be taken into consideration when interpreting the results of our analyses. Despite these limitations, the participation of different cohorts is an important part of the richness of the project and will enable both testing for the heterogeneity and provision of important information on the generalizability of the findings.

## Can I get hold of the data? Where can I find out more?

The NVP Genetics Consortium is actively searching for new groups interested in joining this common effort to elucidate the causes of NVP and HG. The Consortium does not require researchers to share raw data and, as such, the Consortium does not hold raw data that can be accessed by other researchers. However, the Consortium is very keen to support collaboration and will make results and summary statistics from genome-wide association analyses available from the consortium webpage: [https://genepi.qimr.edu.au/staff/sarahMe/nvp/index.html]. Interested researchers should contact Dr Sarah Medland, whose details are provided in the contact author information.

NVP Genetics Consortium in a nutshell

- The NVP Genetics Consortium is an open collaborative network of researchers aiming to study the risk factors, with a special focus on the genetic factors, for the development of nausea and vomiting during pregnancy (NVP), commonly known as morning sickness.
- Consortium efforts started in 2013. The Consortium currently brings together cohorts from Australia, Finland, Spain and the UK, including data from 16 756 women of whom 80% reported NVP, and data from 9074 women who have suffered extreme NVP from a Danish population registry.
- Some cohorts have existing data available on NVP and decided to join the Consortium, whereas others specifically collected data with this aim. Some have planned follow-ups to enrich the available data. Age at data collection ranges between 13 and 78.
- The NVP Genetics Consortium is collecting data of presence, duration and severity of NVP, demographic and health-related measures. Some of the samples are already genotyped.
- The NVP Genetics Consortium is actively searching for new collaborations. Summary data from GWAS will be made available at the Consortium website.

## Funding

This work was supported by several funding agencies. QIMR Berghofer data collections have been funded by the Australian Research Council [grant numbers A79600334, A79906588, A79801419, DP0212016, DP0343921] and the National Health and Medical Research Council Project Grant [grant numbers 241944, 389875, 552485, 552471, 1031119, 1049894, 1084325]. The GSA data collections have been funded by the Stiftelsen för Åbo Akademi Foundation [grant number 21/22/05] and the Academy of Finland [grant numbers 138291, 210298, 212703]. The MTR is funded by Fundación Séneca-Regional Agency for Science and Technology, Murcia, Spain [grant numbers 03082/PHCS/05, 08633/PHCS/08, 15302/PHCS/10] and the Spanish Ministry of Science and Innovation [grant numbers PSI2009–11560, PSI2014-56680-R]. The UK Medical Research Council and the Wellcome Trust [grant number 102215/2/13/2] and the University of Bristol provide core support for ALSPAC. This work was also supported by Fundación Séneca-Regional Agency for Science and Technology, Murcia, Spain [19151/PD/13 to L.C.C.], the Medical Research Council [MR/J012165/1 to L.P.] and the National Institute of Mental Health [R01MH104468 to T.M.O. and M.L.]. LP works in a unit funded by the Medical Research Council (MC_UU_12013/4).
